# Mathematical Model of the Spread of Hantavirus Infection

**DOI:** 10.3390/pathogens12091147

**Published:** 2023-09-08

**Authors:** Juan Pablo Gutiérrez-Jara, María Teresa Muñoz-Quezada, Fernando Córdova-Lepe, Alex Silva-Guzmán

**Affiliations:** 1 Centro de Investigación de Estudios Avanzados del Maule (CIEAM), Vicerrectoría de Investigación y Postgrado, Universidad Católica del Maule, Talca 3480112, Chile; 2 School of Public Health, Faculty of Medicine, Universidad de Chile, Avenida Independencia 939, Santiago 8320000, Chile; mtmunoz@uchile.cl; 3 Facultad de Ciencias Básicas, Universidad Católica del Maule, Avenida San Miguel 3605, Talca 3480112, Chile; fcordova@ucm.cl; 4 Maule Regional Ministerial Health Secretariat, Talca 3480112, Chile

**Keywords:** hantavirus, mathematical model, zoonotic disease

## Abstract

A mathematical epidemiological model incorporating the mobility of rodents and human groups among zones of less or major contact between them is presented. The hantavirus infection dynamics is expressed using a model type SEIR (Susceptible-Exposed-Infectious-Removed), which incorporates the displacement of the rodent and the human, between the urban and rural sector, the latter being subdivided in populated and non-populated. The results show the impact that rodent or human displacement may have on the propagation of hantavirus infection. Human mobility is more significant than rodents in increasing the number of hantavirus infection cases. The results found may be used as a reference by the health authorities to develop more specific campaigns on the territorial dynamics of the rodent, attend to the mobility of humans in these territories, mainly agricultural and forestry workers, and strengthen control-prevention actions in the community, to prevent future outbreaks that are fatal.

## 1. Introduction

Hantavirus cardiopulmonary syndrome (HCPS) is one of the zoonotic viral diseases caused by the family of viruses of the order *Bunyavirales*, within which are the Sin Nombre virus (SNV) and the Andes virus (ANDV) [1], which are the most common zoonotic agents that cause HCPS and are found mainly in rodents such as *Peromyscus maniculatus* (deer mouse) or *Oligoryzomys longicaudatus* (long-tailed mouse) [2,3]. Only in Argentina and Chile are HCPS associated with ANDV infection through the long-tailed mouse [2]. Because of the genus *Hantavirus*, in addition to HCPS, other hantaviruses can cause hemorrhagic fever with renal syndrome (HFRS).

HCPS occurs mainly in the Americas, while HFRS occurs in Asia and Europe [4]. HCPS was discovered in the United States in 1993, when SNV was identified, with an initial mortality of 50%, and to date there is no cure [2,5,6]. At first, it was called hantavirus pulmonary syndrome; however, it was redefined as hantavirus cardiopulmonary syndrome because the leading cause of death is myocardial depression [7].

HCPS has a mortality rate of 50% of cases infected by ANDV [8], with the lethality of this hantavirus disease between 30 and 60% [5]. The high lethality can be correlated with the short intervention period since, on average, one has three days between the first symptoms and the first consultation, and two more days between the first consultation and death; that is to say, it is very brief [6]. Furthermore, there is a relationship between socioeconomic level and this indicator, since more significant economic vulnerability increases the probability of death [5].

Transmission of the virus from rodent to humans occurs primarily through inhalation of viral particles found in the fluids of this infected rodent, such as urine, faeces, or saliva [9,10]. This contagion occurs in work activities (particularly forest workers and farmers), or recreational or domestic scenarios [2,11,12,13]. There are also other types of infection, but these are more isolated cases, such as bites or by eating an infected rodent. There are cases of person-to-person transmission, occurring mainly in Argentina and Chile [9,14,15,16]. Still, these have been isolated cases, except for what happened in Argentina between November 2018 and February 2019, resulting in 34 confirmed cases of contagion of the virus, ANDV among people, and 11 deaths [15].

The number of people infected each year varies; this can occur due to the ecosystem variations of recent years produced by climate change, such as forest fires, among others, increasing the frequency of high-impact events [2]. In addition, the flowering of the *Chusquea quila* and the *Chusquea colihue* (the main food of the rodent) has boosted the increase in the reservoir population [17].

The countries in America with the highest incidence of hantavirus infection (HI) correspond to Brazil, Argentina, and Chile [2]. In Argentina and Chile, the main reservoirs are found among mice: *Oligoryzomys longicaudatus, Abrothrix olivaceus*, and *Akodon longipilis*; the first of these was found with a more infected population in these two mentioned countries [18]. It should be noted that in Argentina in 2021 a new reservoir was found, *Scapteromys aquaticus*, further expanding the diversity of reservoirs [19]. In Brazil, there are many hantavirus reservoirs; *Oligoryzomys nigripi* and *Necromys lasiurus* are some of them [20].

Most mathematical models study the dynamics of the hantavirus among rodents [21,22,23,24,25,26]. Some distinguish between males and females [21,23]; others compare direct with indirect transmission, involving demographic, environmental, or seasonal variables [24,25,27]; and in [28], they predict the territorial distribution of infected rodents. However, there are models focused on cases of HI in humans; for example, in [29], the human population is divided into agricultural workers and others, while in [30], with a statistical approach, the article projects possible future cases according to the environmental variable. Our study presents a mathematical-epidemiological generalist model representing the territorial dynamics between humans and rodents (main novelty). It aims to analyze this territorial distribution impact on the disease (HI) spread in the human co-inhabiting population.

To express the dynamics of the disease among rodents, we rely on a compartmental model type SEIR (Susceptible-Exposed-Infectious-Removed) [31]. At the same time, for the sanitary condition of humans toward infection, there are two states: susceptible or infected (non-infectious); although there are reported cases of contagion among people, these are particular cases and more studies are needed, so for simplicity of the model, transmission between people will not be considered, only their mobility.

Next, relevant data on HI propagation will be presented (Section 2.1), as well as the mathematical model of HI together with the study of the proposed system (Section 2.2), different numerical simulations based on the exposed model (Section 3), and finally (Section 4), the conclusions obtained from the research will be discussed.

## 2. Materials and Methods

### 2.1. Relevant Data

According to the Pan American Health Organization (PAHO), the cases reported by HI until 2017 in America correspond to 800 in North America, 269 in Central America, and 5243 in South America [2].

The most significant number of individuals with HI are reported in South America, mainly concentrated in the countries of Brazil (2032), Argentina (1350), and Chile (1028) [2]. Considering that the existing population of the countries mentioned above in 2017 is 209.3, 44.27, and 18.05 million people, respectively, the prevalence of HI remains 0.971, 3.015, and 5.695 cases per 100,000 inhabitants. Therefore, Chile has the highest prevalence rate in Latin America.

In Chile, in a study carried out by Reyes and Ferrés [5], they reviewed 997 records published by the Ministry of Health through the Bulletin Notification of Obligatory Declaration Diseases (ENO) between 1996 and 2016, including retrospective cases corresponding to 1975 and 1993 to 1995; it is evident that the highest number of HI cases, possibly from the long-tailed mouse to humans, is in rural residents; see Table 1.

According to a report delivered by the Chilean Ministry of Health, from 2017 to 18 March 2022 [32], the main risk factor continues to be people with rural residence, with a considerable increase since 2019 in the risk factor “Others”, that is, contagion occurs in a different place than usual (see Table 2).

In Chile, the spread of HI occurs mainly in rural areas near the Andes’ foothills; the regions most affected are those of the central and southern zone between Valparaiso and Aysen [9]. By 2019, 70 cases were observed, with a national incidence rate of 0.4%, where the Maule region presented the greatest number of HI cases (*n* = 15), see Table 3.

Considering the above information [5,9,32], the Chilean Ministry of Health was requested to provide historical data for the last decade on HI cases in the Maule region, which are presented below (Table 4).

As shown in Table 4, there is an increase in HI cases, with a 33% fatality rate.

**Figure 1:**
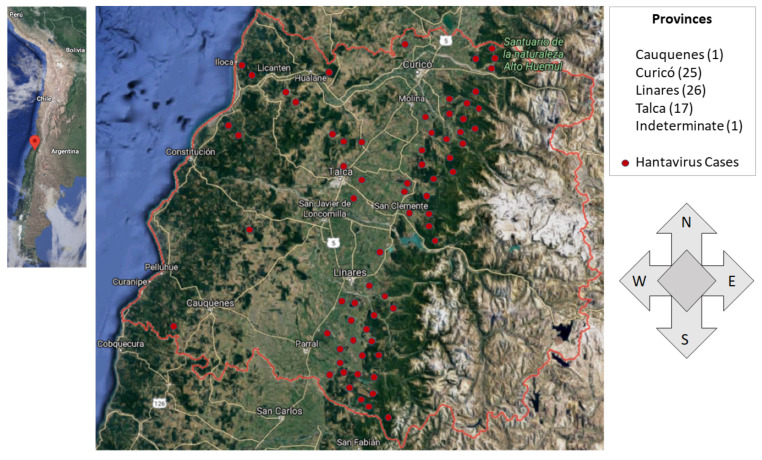
Total of 70 hantavirus infection cases between the years 2010 and 2019 in the Maule Region, Chile. Points in red represent a localization estimate of the georeferenced cases via Google Maps. Inner satellite image credit: Google Maps, 2021 (https://www.google.com/maps (accessed on 31 January 2022)).

The distribution of cases reported in the Maule Region between 2010 and 2019 is concentrated in the east of the region, that is, in the sectors near the Andes (see Figure 1), but there are also cases in coastal areas (west of the region).

### 2.2. Model

The dynamics of hantavirus have been studied among rodents in several mathematical models [21,23,26], which are expressed by the SEIR type (Susceptible, Exposed, Infectious, Removed). In our model, we will be based on this relationship.

As mentioned in the Introduction, there are confirmed cases of human-to-human transmission of HI [9,14,15]. Although they are very relevant data to consider, in our study, we will avoid contagion between people because we believe that more studies are needed to affirm this assertion. However, incorporating the human-to-human transition into the model will be left for work in other investigation in the not distant future.

HI has an incubation period of one to six weeks [2]. Since we are assuming that the disease is not transmissible between people, for simplicity of the model, we will express only two states about the human health condition: Susceptible (*S*) and Infected (*D*). This last state (*D*) refers to the cumulative cases of people who have had HI. It is important to note that there is no authorized treatment or vaccine to date, and the people who manage to survive the infection do so due to the antibodies they generate, achieving immunity to the virus [33,34].

About the territorial dynamics, we will consider two sectors: Urban (*u*) and Rural. By urban sector, we mean the group of houses whose population exceeds 2000 inhabitants, or if the population is concentrated between 1001 and 2000 inhabitants and with 50% or more of its economically active population dedicated to secondary and/or tertiary activities [35]. Otherwise, it is considered a rural sector. In the urban sector (*u*) only the presence of humans will be considered, while in the rural sector the presence of the rodent carrying the infection will also be included. The rural sector will also be subdivided into two: populated (*a*) and non-populated (*f*). In sector (*a*), both humans and rodents will be assumed to be present but with humans’ greater presence, while for the non-populated sector (*f*) only rodents will be assumed to be present. Thus, in summary, the model will have three sectors: Urban (*u*), populated rural (*a*), and non-populated rural (*f*). The assumption of no presence of infected rodents in the urban sector was based mainly on the information provided by [9,32]. Although there are transmission cases in the said locality, these are isolated. The houses where contagions occur are those that, although within the sector, are further away from the general community.

On the human side, tourists and agricultural and forest workers, among others, can be considered outsiders. On the rodent side, it can be considered that, due to food shortage, forest fires, or climate change, rodents found in their natural habitat (rural non-populated, *f*) go to the rural populated sector (*a*). Therefore, we assume a unilateral flow regarding the displacement in rodents, from *f* to *a*, while for humans, it will move between the three sectors. Thus, the abundance of each compartment is summarized in Table 5.

The parameters associated with human mobility between sectors are summarized in Table 6. $\nu_{u}$ and $\nu_{a}$ denote the exit rate from the urban and rural sectors. The fraction of the population that goes to another sector is represented by α, whose parameter has two subscripts meaning the place of departure and destination, reading from right to left. For example, $\alpha_{au}$ is the fraction of the population that lives in the urban sector (*u*) that chooses the populated rural sector (*a*) as its destination. The average time spent by people in the urban and rural sectors populated in other places is denoted by $\tau_{u}$ and $\tau_{a}$, respectively. Regarding the mobility of the rodent (see Table 6), the exit rate (ϕ) is from the non-populated rural sector. The average time that they remain in the populated rural sector is denoted σ. Of the rodents that leave at a rate ϕ, the fraction according to their disease condition is represented by $\lambda_{s}$, $\lambda_{e}$, $\lambda_{i}$, $\lambda_{r}$ depending on whether they are in their state of susceptible, exposed, infectious, or recovered, respectively.

Regarding the parameters associated with the disease (see Table 6), in humans, $\beta_{a}$ and $\beta_{f}$ denote the transmission rate from rodents to humans in populated and unpopulated rural sectors, respectively. For the rodent, β is the rate of transmission between the rodents. The rodent’s average incubation and infection time are 1/δ and 1/γ, respectively.

**Figure 2:**
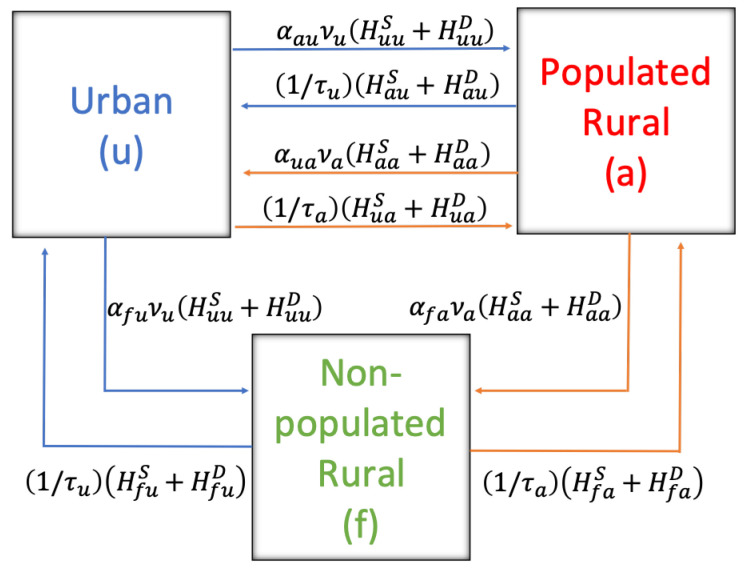
Territorial dynamics of the human being.

To visualize the displacement and possible infections, the respective flow charts are expressed. Figure 2 shows the movement of humans between territories. In blue, the displacement of human residents in the urban sector (*u*) is shown and in red, those in the populated rural sector (*a*).

**Figure 3:**
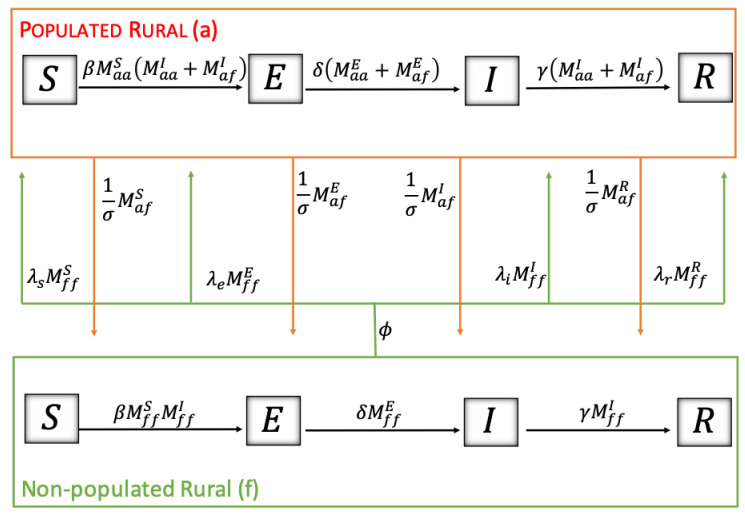
Territorial dynamics of the rodent.

Figure 3 shows the dynamics of the disease among rodents using S-E-I-R states and how resident rodents in the rural non-populated sector (*f*) travel in proportions and for a given time to the rural populated sector (*a*). The movement of the resident rodents of the rural populated sector is red, while those of the non-populated sector are green.

**Figure 4:**
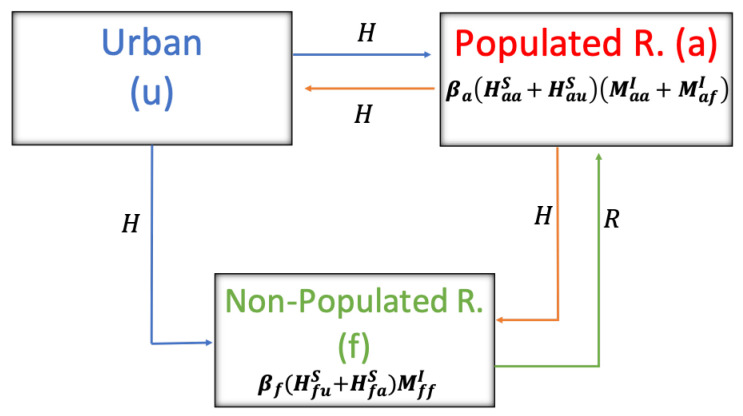
Territorial dynamics between humans and rodents.

Figure 4 shows the interaction between humans and rodents with their respective infection rates, depending on where they are found, either in the populated or non-populated rural sector.

The model is represented by the system of ordinary differential equations expressed in (1).

(1) $$\left\{\begin{array}{c} \begin{array}{cc} {\dot{H}}_{uu}^{S} & =-\nu_{u}H_{uu}^{S}+\frac{1}{\tau_{u}}\left(H_{au}^{S}+H_{fu}^{S}\right)\\ {\dot{H}}_{au}^{S} & =\alpha_{au}\nu_{u}H_{uu}^{S}-\frac{1}{\tau_{u}}H_{au}^{S}-\beta_{a}H_{au}^{S}\left(M_{aa}^{I}+M_{af}^{I}\right)\\ {\dot{H}}_{fu}^{S} & =\alpha_{fu}\nu_{u}H_{uu}^{S}-\frac{1}{\tau_{u}}H_{fu}^{S}-\beta_{f}H_{fu}^{S}M_{ff}^{I}\\ {\dot{H}}_{aa}^{S} & =-\nu_{a}H_{aa}^{S}+\frac{1}{\tau_{a}}\left(H_{ua}^{S}+H_{fa}^{S}\right)-\beta_{a}H_{aa}^{S}\left(M_{aa}^{I}+M_{af}^{I}\right)\\ {\dot{H}}_{ua}^{S} & =\alpha_{ua}\nu_{a}H_{aa}^{S}-\frac{1}{\tau_{a}}H_{ua}^{S}\\ {\dot{H}}_{fa}^{S} & =\alpha_{fa}\nu_{a}H_{aa}^{S}-\frac{1}{\tau_{a}}H_{fa}^{S}-\beta_{f}H_{fu}^{S}M_{ff}^{I}\\ {\dot{H}}_{au}^{D} & =\beta_{a}H_{au}^{S}\left(M_{aa}^{I}+M_{af}^{I}\right)\\ {\dot{H}}_{fu}^{D} & =\beta_{f}H_{fu}^{S}M_{ff}^{I}\\ {\dot{H}}_{aa}^{D} & =\beta_{a}H_{aa}^{S}\left(M_{aa}^{I}+M_{af}^{I}\right)\\ {\dot{H}}_{fa}^{D} & =\beta_{f}H_{fa}^{S}M_{ff}^{I}\\ {\dot{H}}^{d} & =d\{\beta_{a}H_{au}^{S}\left(M_{aa}^{I}+M_{af}^{I}\right)+\beta_{f}H_{fu}^{S}M_{ff}^{I}+\beta_{a}H_{aa}^{S}\left(M_{aa}^{I}+M_{af}^{I}\right)+\beta_{f}H_{fu}^{S}M_{ff}^{I})\}\\ \; & \\ {\dot{M}}_{aa}^{S} & =b_{ma}N_{ya}-d_{m}M_{aa}^{S}-\beta M_{aa}^{S}\left(M_{aa}^{I}+M_{af}^{I}\right)\\ {\dot{M}}_{ff}^{S} & =-\lambda_{s}\phi M_{ff}^{S}+\frac{1}{\sigma }M_{af}^{S}+b_{mf}N_{yf}-d_{m}M_{ff}^{S}-\beta M_{ff}^{S}M_{ff}^{I}\\ {\dot{M}}_{af}^{S} & =\lambda_{s}\phi M_{ff}^{S}-\frac{1}{\sigma }M_{af}^{S}-d_{m}M_{af}^{S}-\beta M_{af}^{S}\left(M_{aa}^{I}+M_{af}^{I}\right)\\ {\dot{M}}_{aa}^{E} & =-d_{m}M_{aa}^{E}+\beta M_{aa}^{S}\left(M_{aa}^{I}+M_{af}^{I}\right)-\delta M_{aa}^{E}\\ {\dot{M}}_{ff}^{E} & =-\lambda_{e}\phi M_{ff}^{E}+\frac{1}{\sigma }M_{af}^{E}-d_{m}M_{ff}^{E}+\beta M_{ff}^{S}M_{ff}^{I}-\delta M_{ff}^{E}\\ {\dot{M}}_{af}^{E} & =\lambda_{e}\phi M_{ff}^{E}-\frac{1}{\sigma }M_{af}^{E}-d_{m}M_{af}^{E}+\beta M_{af}^{S}\left(M_{aa}^{I}+M_{af}^{I}\right)-\delta M_{af}^{E}\\ {\dot{M}}_{aa}^{I} & =-d_{m}M_{aa}^{I}+\delta M_{aa}^{E}-\gamma M_{aa}^{I}\\ {\dot{M}}_{ff}^{I} & =-\lambda_{i}\phi M_{ff}^{I}+\frac{1}{\sigma }M_{af}^{I}-d_{m}M_{ff}^{I}+\delta M_{ff}^{E}-\gamma M_{ff}^{I}\\ {\dot{M}}_{af}^{I} & =\lambda_{i}\phi M_{ff}^{I}-\frac{1}{\sigma }M_{af}^{I}-d_{m}M_{af}^{I}+\delta M_{af}^{E}-\gamma M_{af}^{I}\\ {\dot{M}}_{aa}^{R} & =-d_{m}M_{aa}^{R}+\gamma M_{aa}^{I}\\ {\dot{M}}_{ff}^{R} & =-\lambda_{r}\phi M_{ff}^{R}+\frac{1}{\sigma }M_{af}^{R}-d_{m}M_{ff}^{R}+\gamma M_{ff}^{I}\\ {\dot{M}}_{af}^{R} & =\lambda_{r}\phi M_{ff}^{R}-\frac{1}{\sigma }M_{af}^{R}-d_{m}M_{af}^{R}+\gamma M_{af}^{I}, \end{array} \end{array}\right.$$

where $H^{d}$ corresponds to the number of deaths (cumulative) due to the spread of HI. In addition, $N_{ya}=M_{aa}^{S,E,I,R}$, $N_{yf}=M_{ff}^{S,E,I,R}+M_{af}^{S,E,I,R}$, and $b_{ma}+b_{mf}=d_{m}$. We will assume that the probability of infection to humans in the non-populated rural sector (*f*) is greater than in the populated rural sector (*a*), $\beta_{a}<\beta_{f}$, since in *a*, the presence of humans implies intervention in the landscape, maintenance of more explicit areas, wider paths, and frequent cleaning, among others, in addition to more permanent anthropogenic noise. Therefore, it is plausible to assume that human–rodent contact in the populated rural sector is less likely to be an effective contact compared to the unpopulated rural sector.

It is important to note that the model presented assumes constant rates. In addition, factors or variables that can alter the mobility of humans and rodents impacting hantavirus transmission, such as psychosocial, environmental, risk perception, and intraspecific competition, among others, have been left out. However, including these variables in the model presented opens up various possibilities for future interdisciplinary work.

#### Basic Reproductive Number

One of the central values establishing epidemic threshold conditions is the basic reproductive number, denoted by $R_{0}$. This value determines the “expected number of secondary infectious cases produced by a first infectious in a completely susceptible population” [36,37]. If we consider only the dynamics of the rodents, the system (1) is reduced to 12 equations. Using the next-generation matrix [37], the basic reproductive number of the rodent ($R_{0}^{M}$), which is given by

$$R_{0}^{M}=\frac{\beta \{\sqrt{A}+B\}}{2\left(\gamma +d_{m}\right)\left(\gamma +d_{m}+1/\sigma +\lambda_{i}\phi \right)},$$

where

$$A={\left\{M_{aa}^{S^{*}}\left(\gamma +d_{m}+1/\sigma +\lambda_{i}\phi \right)-M_{ff}^{S^{*}}\left(\gamma +d_{m}+1/\sigma \right)+M_{af}^{S^{*}}\left(\gamma +d_{m}+\lambda_{i}\phi \right)\right\}}^{2}+{\left\{2M_{ff}^{S^{*}}\left(1/\sigma \right)M_{af}^{S^{*}}\lambda_{i}\phi \right\}}^{2}$$

and $B=M_{aa}^{S^{*}}\left(\gamma +d_{m}+1/\sigma +\lambda_{i}\phi \right)+M_{ff}^{S^{*}}\left(\gamma +d_{m}+1/\sigma \right)+M_{af}^{S^{*}}\left(\gamma +d_{m}+\lambda_{i}\phi \right)$, with $M_{xy}^{S^{*}}$ for x,y∈{a,f}, corresponds to the disease-free equilibrium.

About human dynamics, since we do not consider contagion among humans, nor from humans to rodents, it is not possible to determine the basic reproductive number associated with the human population. However, an interpretative approach to the spread of the disease in humans will be established.

The basic reproductive number, in general, depends on three factors: infectious period, contact rate, and probability of transmission [36]. From our model expressed in (1), in HI the infectious period depends on the rodent, while the contact rate and transmission probability depend on both (human and rodent). As mentioned above, we can deduce that the HI transmission (*℘*) can be expressed by: $℘={℘}_{a}+{℘}_{f}$, where ${℘}_{a}$ and ${℘}_{f}$ correspond to the infections carried out in the populated and non-populated rural sectors, respectively. For each of them, (${℘}_{a}$ and ${℘}_{f}$) can be subdivided into the contagion caused by rodents that inhabit the place and by foreign rodents (that enter the sector). Thus, ${℘}_{a}$ without considering the entry and exit of rodents, the average time a rodent remains infectious is given by $1/(\gamma +d_{m})$, while the contact rate and transmission probability are given in combined form by

(2) $$\beta_{a}*\left\{\alpha_{au}\nu_{u}H_{uu}^{S}\left(0\right)+\left(1/\tau_{a}\right)\left(H_{ua}^{S}\left(0\right)+H_{fa}^{S}\left(0\right)\right)+\left(1-\nu_{a}\right)H_{aa}^{S}\left(0\right)\right\},$$

where $\alpha_{au}\nu_{u}H_{uu}^{S}\left(0\right)$ represents the percentage of humans entering from the urban sector to the rural populated sector, $\left(1/\tau_{a}\right)\left(H_{ua}^{S}\left(0\right)+H_{fa}^{S}\left(0\right)\right)$ expresses the percentage of humans returning to the rural populated sector from the other sectors, and $\left(1-\nu_{a}\right)H_{aa}^{S}\left(0\right)$ symbolizes the percentage of humans remaining in the rural populated sector. For the contagion produced by the entry and exit of rodents, the average time that a rodent remains infectious is given by $1/(\gamma +d_{m}+1/\sigma )$, while the contact rate and transmission probability is similar to (2) except that the rates associated with the exit of the rodent from the non-populated rural sector (ϕ) are involved, with their respective infectious rodent fraction ($\lambda_{i}$), leaving

(3) $$\beta_{a}*\lambda_{i}*\phi *\left\{\alpha_{au}\nu_{u}H_{uu}^{S}\left(0\right)+\left(1/\tau_{a}\right)\left(H_{ua}^{S}\left(0\right)+H_{fa}^{S}\left(0\right)\right)+\left(1-\nu_{a}\right)H_{aa}^{S}\left(0\right)\right\}.$$

Therefore,

(4) $${℘}_{a}=\left(\frac{\beta_{a}}{\gamma +d_{m}}+\frac{\beta_{a}\lambda_{i}\phi }{\gamma +d_{m}+1/\sigma }\right)H_{a}^{*},$$

with $H_{a}^{*}=\alpha_{au}\nu_{u}H_{uu}^{S}\left(0\right)+\left(1/\tau_{a}\right)\left(H_{ua}^{S}\left(0\right)+H_{fa}^{S}\left(0\right)\right)+\left(1-\nu_{a}\right)H_{aa}^{S}\left(0\right)$.

Similarly, ${℘}_{f}$ is obtained, leaving

(5) $${℘}_{f}=\left(\frac{\beta_{f}}{\gamma +d_{m}}+\frac{\beta_{f}\left(1/\sigma \right)}{\gamma +d_{m}+\lambda_{i}\phi }\right)H_{f}^{*},$$

with $H_{f}^{*}=\alpha_{fu}\nu_{u}H_{uu}^{S}\left(0\right)+\alpha_{fa}\nu_{a}H_{aa}^{S}\left(0\right)+\left(1-\left(1/\tau_{u}\right)\right)H_{fu}^{S}\left(0\right)+\left(1-\left(1/\tau_{a}\right)\right)H_{fa}^{S}\left(0\right)$.

Therefore, *℘* is given by,

(6) $$℘=\left(\frac{\beta_{a}}{\gamma +d_{m}}+\frac{\beta_{a}\lambda_{i}\phi }{\gamma +d_{m}+1/\sigma }\right)H_{a}^{*}+\left(\frac{\beta_{f}}{\gamma +d_{m}}+\frac{\beta_{f}\left(1/\sigma \right)}{\gamma +d_{m}+\lambda_{i}\phi }\right)H_{f}^{*}.$$

Equation (6) can be rewritten by:

(7) $$℘=\frac{\beta_{a}}{\gamma +d_{m}}\left(1+\frac{\lambda_{i}\phi \left(\gamma +d_{m}\right)}{\gamma +d_{m}+1/\sigma }\right)H_{a}^{*}+\frac{\beta_{f}}{\gamma +d_{m}}\left(1+\frac{\left(1/\sigma \right)\left(\gamma +d_{m}\right)}{\gamma +d_{m}+\lambda_{i}\phi }\right)H_{f}^{*}.$$

In this way, it can be explicitly observed how the mobility parameters of the rodent (ϕ and σ) and those of the human (α’s, ν’s, and τ’s) influence the propagation of HI.

For the cases where there is no mobility of the rodent (ϕ=0 and 1/σ=0), the expected is obtained, which is $\sum_{x}\left\{\beta_{x}/\left(\gamma +d_{m}\right)\right\}H_{x}^{*}$ with x∈{a,f}.

Based on the definition of the basic reproductive number [36,37] and others that were based on this [38], the *℘*-value can be defined as “the expected number of infected human cases, produced by an infectious rodent, in a population of susceptible humans”.

## 3. Results

To express the dynamics of the model presented, the numerical simulations (using the ode45 function of MATLAB) will be carried out with the data associated with Chile, particularly with the Maule region, whose total population, according to the 2017 census, corresponds to 1,044,950 inhabitants, where approximately 70% of the population lives in the urban sector and 30% in the rural sector [35].

**Figure 5:**
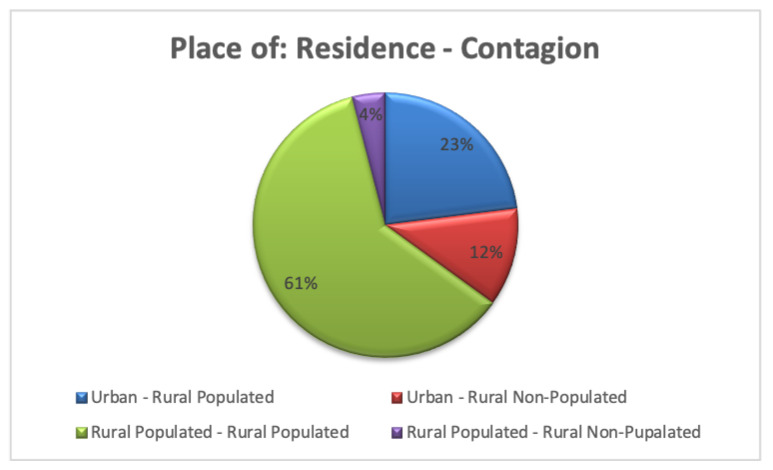
Total of 70 hantavirus infection cases, between 2010 and 2019 in the Maule region, Chile.

According to the data provided by the SEREMI of Health Maule region [9], a region located in south-central Chile, we can classify the residence and the place of infection among the three sectors of our model: Urban (*u*), Rural populated (*a*), and Rural non-populated (*f*), which shows that the greatest cases occur among people who live in the rural populated sector (61%), followed by people who live in the urban sector and are infected in the rural populated (23%). They are followed by people living in the rural populated and urban sectors who are infected in the rural unpopulated sector, with 12% and 4%, respectively; see Figure 5.

The numerical values associated with the different rates presented in the model have been extracted from other investigations [21,23,26,29]. Based on the information provided in Figure 5 (see Table 6), these have been chosen to obtain a projection treating to incorporate more realism.

**Figure 6:**
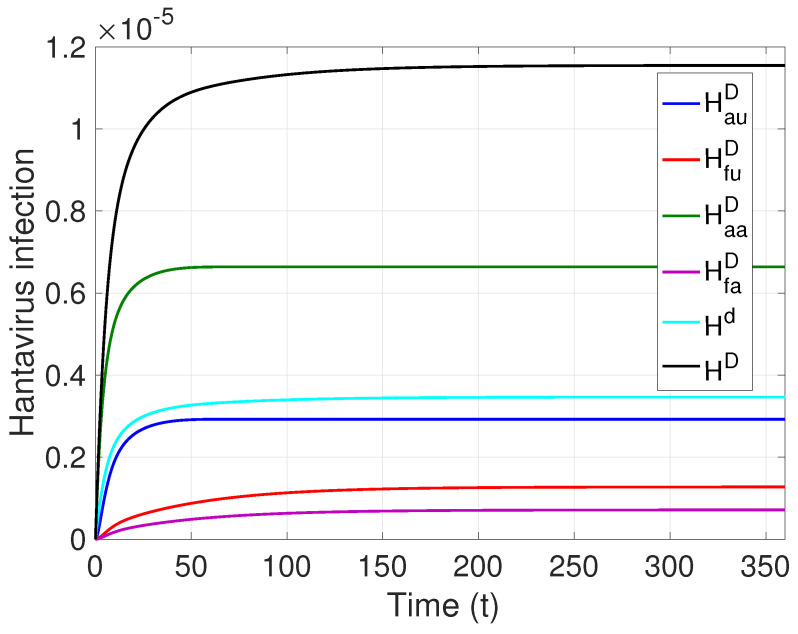
Hantavirus infection in the Maule region, Chile $H^{D}=H_{au}^{D}+H_{fu}^{D}+H_{aa}^{D}+H_{fa}^{D}$; $R_{0}^{M}=1.0352$, $℘=9.6965\times 10^{-5}$.

Thus, with the chosen rates, we obtain that (see Figure 6) the percentage of the human population infected by HI during a year amounts to $1.155\times 10^{-5}$, that is, approximately 0.0012% of the population, which is expressed in the Maule region as a total of 12 infected people (value according to the data provided in Table 4), and which, when distributed among the sectors of the model, is 58, 25, 11, and 6 (%) for $H_{aa}^{D}$, $H_{fu}^{D}$, $H_{au}^{D}$, and $H_{fa}^{D}$, respectively (values very close to those provided in Figure 5). Mortality corresponds to $0.397\times 10^{-5}$, which is equivalent to 30% of the infected population (four cases).

In what follows, we proceed to visualize the impact that mobility parameters have on the development of the disease.

**Figure 7:**
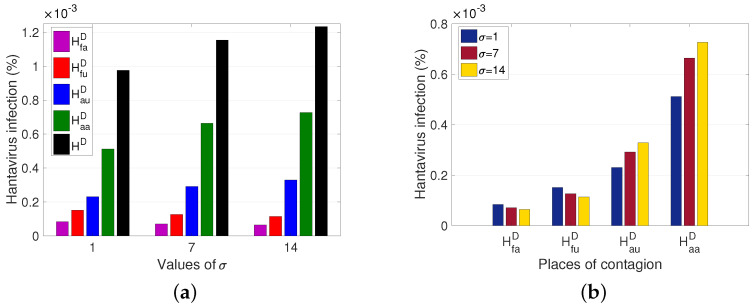
Variation of total hantavirus infection cases concerning the average time a rodent remains an outsider. $H^{D}=H_{au}^{D}+H_{fu}^{D}+H_{aa}^{D}+H_{fa}^{D}$. (**a**) Hantavirus infection cases according to a place of infection for different σ. (**b**) Hantavirus infection cases after an increase in σ, for each place of infection. For σ=1, 7, 14, it has that $R_{0}^{M}=1.0406\;,1.0252\;,1.0333$ and $℘=1.0626\times 10^{-4},\;9.6965\times 10^{-5},\;9.6215\times 10^{-5}$, respectively.

The average time that a rodent from the non-populated rural sector remains in the populated rural sector (σ) is a factor to consider. The longer the rodent stays as an outsider, the more HI cases increase for people living in ($H_{aa}$) or traveling to the rural sector ($H_{au}$), while the cases associated with infection in the rural sector ($H_{fa}^{D}$ and $H_{fu}^{D}$) decrease (see Figure 7b). It is also observed that the total number of HI cases increases by approximately 0.0003% of the population, after the increase of σ between 1 and 14 days (see Figure 7a).

**Figure 8:**
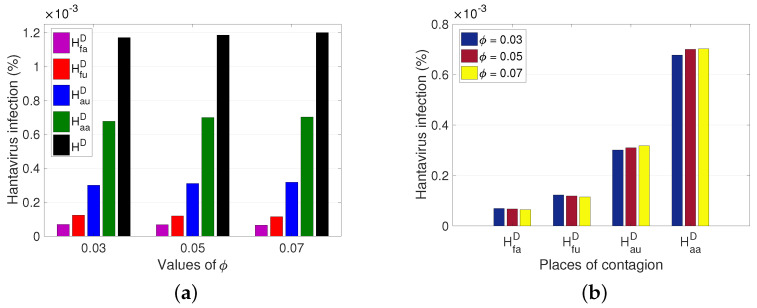
Variation of the total number of hantavirus infection cases concerning the exit rate of the group of rodents living in the rural non-populated sector. $H^{D}=H_{au}^{D}+H_{fu}^{D}+H_{aa}^{D}+H_{fa}^{D}$. (**a**) Hantavirus infection cases according to a place of infection for different ϕ. (**b**) Hantavirus infection cases after an increase in ϕ, for each place of infection. For ϕ=0.03 ,0.05, 0.07, it has that $R_{0}^{M}=1.0205\;,1.0062\;,0.9923$ and $℘=9.7174\times 10^{-5},\;9.7384\times 10^{-5},\;9.7596\times 10^{-5}$, respectively.

Another relevant factor is the proportion of the rodent population living in the unpopulated rural sector that goes into the populated rural sector (ϕ). Figure 8 shows an effect similar to that of Figure 7, since although there is a decrease in cases in the non-populated rural sector, the number of cases in the populated rural sector increases, and the total number of cases increases.

**Figure 9:**
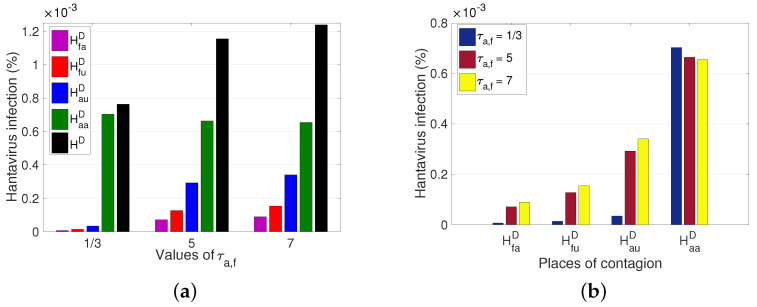
Variation of total hantavirus infection cases concerning the average time a rodent remains an outsider. $\tau_{a,f}$ indicates that $\tau_{a}=\tau_{f}$. $H^{D}=H_{au}^{D}+H_{fu}^{D}+H_{aa}^{D}+H_{fa}^{D}$. (**a**) Hantavirus infection cases according to a place of infection for different $\tau_{a,f}$. (**b**) Hantavirus infection cases after an increase in $\tau_{a,f}$, for each place of infection. For $\tau_{a,f}=1/3,\;5,\;7$, it has that $R_{0}^{M}=1.0352$ for all and $℘=4.6565\times 10^{-4},\;9.6965\times 10^{-5},\;1.1856\times 10^{-4}$, respectively.

In addition to the dynamics of the reservoir’s movement, it is relevant to observe the effect that the movement of the human has in different scenarios by varying the average time spent as an outsider between the different sectors. Three cases are presented; the first corresponds to people who leave their sector and stay eight hours in another sector, that is, $\tau_{a,f}=1/3$ (where $\tau_{a,f}$ expresses that $\tau_{a}=\tau_{f}$), followed by the second case, where they stay 5 days (from Monday to Friday), and finally, the third case, which is 7 days (full week). From Figure 9a, one can see the significant increase in the total number of HI cases in the population, after the variation of $\tau_{a,f}$, increased by approximately 0.0005% of the population. Concerning the number of HI cases per sector, we explicitly observe how the number of cases is altered after the variation of $\tau_{a,}$ (see Figure 9b).

## 4. Discussion and Conclusions

The dynamics of HI transmission were modeled by ordinary differential equations, incorporating the mobility of humans and rodents in three sectors: urban, populated rural, and non-populated rural, the latter two being the territories where infection occurs. To build the model, we have relied on previous compartmental models that study these dynamics without territorial mobility, the main novelty of our work. The data that feed our model were mainly from the cases reported in Chile and other studies previously carried out in different parts of the world. The scarcity of mobility information is one of the main limitations. However, the generality of our model can provide considerable qualitative results that, in a novel way, consider the mobility of humans and rodents, contributing to the literature and informing the guidelines in public health decision-making.

From the background consulted on the presence of the lethal HI disease in the Maule region and based on the existing literature, we mathematically modeled the territorial dynamics between humans and rodents, evidencing the impact on the spread of HI that can occur with increased human mobility.

Several variables are identified in the recent literature associated with the prevalence of HI and its infection through the rodent. For example, forest fires, high temperatures, and droughts are identified as precipitating factors for the increased prevalence of zoonoses [39,40]. A study conducted in a boreal forest in Sweden, which suffered a large-scale fire in 2006, determined a high risk of infection of Puumala orthohantavirus in areas close to fires due to the mobility and resistance of the rodent in the affected habitats; however, the risk was found to be even higher in non-burned forests [39].

In the Maule region, Chile, since 2017, there have been a series of large forest fires that have mainly affected coastal areas or the central valley, with fewer reported cases of hantavirus infection. However, in the summer of 2020, a forest fire occurred in the Agua Fría sector in the municipality of Molina (Andes foothills sector), which affected about 13800 hectares, an event that could affect the displacement of rodents from their habitat in non-populated rural areas due to the possible absence of food to populated places, increasing the interaction between rodents and humans. However, due to the COVID-19 pandemic, people’s mobility has been reduced by the prevention measures imposed by the Chilean government (mainly quarantines and sanitary cordons), so there was no increase in HI cases [32,41,42].

On the other hand, the change in land use from native forests to agricultural and forest lands as well as high temperatures in humid climates are identified as relevant factors in the interaction between agricultural workers and the rodent and, therefore, in the spread of hantavirus [43]. A study in the Atlantic forest [44] indicates that forest restoration could reduce the possibility of HI transmission by 45%. In [45], they compared the exclusion of terrestrial mammalian predators and the degradation of the Interior Atlantic forest, showing that seasonality and landscape composition play a fundamental role in the prevalence of rodent reservoirs; in contrast, the exclusion of predators had little influence on the rodent population.

A study conducted in Chile [46], during 19 years of sampling eleven rodent species, evaluated how ecology and geography influence host and viral dynamics in areas associated with HI cases in that country, finding that the main ANDV reservoir is *O. longicaudatus* with an intraspecific seroprevalence of 6.5%. They also point out the need for research on rodents’ social and behavioral interactions, highlighting the integration of ecological understanding of the host and pathogens, spatial and temporal surveillance, epidemiology, and public health agencies, as fundamental to understanding the transmission of the virus to humans. Another study conducted in Patagonia, Argentina [47] indicates that the high relative abundance of *O. longicaudatus* in an unstable community associated with peridomestic environments favors intraspecific contact, leading to a higher probability of virus transmission. Thus, our study integrates, in the proposed mathematical model, ecology (rodent habitat), epidemiology (virus transmission), and territorial dynamics (mobility between urban, rural populated, and non-populated sectors) to contribute from this discipline to the description of virus transmission to humans.

A Bayesian analysis carried out with the expansion of sugar cane and the changes of temperature in Sao Paulo and the risk of hantavirus infection, using historical databases between 2000 and 2010 [48], demonstrated that the presence of hantavirus cardiopulmonary syndrome was strengthened by the combination of the effects of climate change associated with the increase in temperatures and the transformation of the rodent’s natural habitat in sugar cane cultivations, evidencing similar conditions reported by another study carried out in China [49] where high temperatures and oscillations of precipitation effected an increase in some types of vegetation that develop in humid areas, influencing the reproduction of the rodent and the mechanism of virus transmission. This, added to the mobility of workers in the rodent territory transformed into an agricultural or forestry crop, increases the risk of spreading hantavirus and the possibility of developing HCPS.

The long-tailed mouse lives less than 2000 m above sea level. In the Maule region, the vegetation and the climate in the Foothills of the Andes mountain range, where the sclerophyllous forest develops with humid zones and high temperatures in the summer, is a suitable habitat for the rodent. Moreover, the population of rodents increases in years that (due to the effect of the Niño current with southern oscillation) there is high pluviometry, which generates an abundance of vegetation and food [48,49]. In these years, population abundance promotes the competition for the territory and the displacement to populated sites. So, the displacement to places where humans live to seek sustenance increases, as does HI risk.

According to the analyzed results, the mobility of people going to non-populated sectors has increased, mainly to the realization of camping and excursions in places not predestined for this type of activity, which could increase the interaction between rodents and humans [50].

Human activities affect natural systems, and global environmental changes put people’s health at risk [51]. In addition, the mobility of people affects not only acute diseases but also chronic ones [52]. On the other hand, it is known that if there is an increase in infected rodents, the possibility of infected humans increases [53,54], which can be attributed to the invasion of humans into the rodent’s habitat. Therefore, people’s mobility and behavior, such as, for example, the absence of self-care behaviors to avoid the spread of hantavirus, impacts the development of zoonotic diseases. From the results of this study, a significant increase in HI cases is exposed after the mobility of people towards sectors with the presence of rodents—transmitters of infection.

One of the main novelties presented in this work is the proposed HI transmission expression (*℘*), which explicitly shows how the rates associated with mobility between different sectors affect the contagion rate from rodents to humans.

From the results obtained, we observed the impact of human and rodent mobility on the spread of HI (Figure 7, Figure 8 and Figure 9). The average time an individual stays in the unpopulated rural sector (Figure 9) is more determinant in HI than the average time a rodent remains in the populated rural sector (Figure 7) and the rodent mobility flow (Figure 8). Therefore, there is a greater risk for the populated rural population due to the proximity of unpopulated rural areas and the type of work activities carried out, ratifying the results of other studies and records, which affirm that the most affected population are the inhabitants of the rural sector and agricultural and forestry workers over the occasional visitor. However, due to the increase in outdoor recreational activities, such as camping, excursions, and others, in non-populated areas, the interaction between rodents and people could increase, leading to a significant increase in cases. Therefore, if prevention and control measures are not established, epidemic outbreaks may occur, which can be fatal due to the high virus mortality rate.

The proposed model is a helpful tool that allows decision-makers, especially in the health and housing areas, to understand the evolution of the phenomenon under different scenarios; therefore, it is essential first to establish the epidemiological, social, and geographical characteristics underlying HI to apply the model and generate strategies. However, based on the data we collected from the Chilean territory we modeled, we recommend, in the first instance, active surveillance strategies for rodent control, public education and awareness, personal and household hygiene, safe food storage, prevention in open spaces, improvement of housing infrastructure, timely medical care, research and monitoring, and intersectoral work between the ministries of health, housing, environment, and other relevant entities to comprehensively address the prevention of HI [55].

In addition to the above, it is essential to avoid constructing houses near nature reserves in risk areas where rodents live [56,57]. In this, it is vital to conduct comprehensive risk assessments that determine the presence of rodents, history of hantavirus cases, and other environmental and epidemiological factors that are sufficient causes of the disease; involve the local community in decision-making on urban development and the protection of natural areas [58] that promote sustainable urban planning which considers the safety of the wild regions and the health of the population [59]; and strengthen legislation and policies that support the prevention, regulation, and restriction of new housing construction in areas identified as high-risk for HI.

Finally, constant surveillance should also be carried out in the rodent’s territory, especially in natural disasters, forest fires, droughts, and adverse effects of climate change. Thus, by having this information and the effort of preventive measures, new cases of HI in humans would be avoided, and given its high lethality, lives would be saved in sectors where there were no cases of hantavirus.

## Figures and Tables

**Table 1:** Confirmed cases of hantavirus infection in Chile until 2016 [5].

Risk Factor	Cases	Percentage (%)	Lethality (%)
Rural Resident	377	77.3	34.2
Agricultural Worker	172	46.6	35.5
Forest Worker	45	14.6	47.7
Excursion	119	34.3	30.8
Exposure in an urban area	43	7.5	37.2

The percentages are over the total of confirmed cases of hantavirus infection in Chile, and some cases present more than one risk factor.

**Table 2:** According to the risk factor, a total number of confirmed cases of hantavirus infection in Chile from 2017 to 18 March 2022 [32].

Risk Factor	2017	2018	2019	2020	2021	2022
Rural resident	50	18	31	20	20	3
Agricultural or forestry worker	31	12	23	14	10	1
Excursion	20	10	17	3	7	1
Contacting a case	5	2	11	7	3	0
Others	17	9	19	12	12	1

Some cases present more than one risk factor.

**Table 3:** The number of cases and incidence rates of hantavirus infection, by region of occurrence of the infection 2017–2020 [9].

Region	Year 2020		Year 2019		Year 2018		Year 2017	
	**Cases**	Incidence *	**Cases**	Incidence *	**Cases**	Incidence *	**Cases**	Incidence *
Valparaíso	0	0.0	2	0.1	0	0.0	1	0.1
Metropolitana	0	0.0	2	0.03	2	0.0	5	0.1
O’Higgins	1	0.1	1	0.1	5	0.5	4	0.4
Maule	3	0.3	15	1.4	3	0.3	7	0.7
BioBío	1	0.1	7	0.4	8	0.4	18	0.8
Ñuble	3	0.6	8	1.6	8	0.4	18	0.8
Araucanía	4	0.4	9	0.9	2	0.2	16	1.6
Los Ríos	5	1.2	12	2.9	1	0.2	10	2.4
Los Lagos	8	0.9	11	1.3	8	0.9	19	2.2
Aysén	3	2.8	1	0.9	2	1.8	2	1.8
Magallanes	0	0.0	0	0.0	0	0.0	0	0.0
In study **	2		2		1		10	
Total	30	0.2	70	0.37	33	0.2	91	0.5

* Rates per hundred thousand inhabitants. ** Cases under study or undetermined of the probable place of infection.

**Table 4:** Confirmed cases of hantavirus infection in the Maule Region, Chile. Between 2010 and 2019. Information provided by the Chilean Ministry of Health.

Year	Cases	Incidence ${}^{*}$	Deceased	Lethality (%)
2010	11	1.09	2	18
2011	5	0.49	1	20
2012	4	0.39	2	50
2013	9	0.88	3	33
2014	2	0.19	0	0
2015	6	0.58	3	50
2016	9	0.86	1	11
2017	6	0.57	2	33
2018	3	0.28	1	33
2019	15	1.37	5	33

* per hundred thousand inhabitants.

**Table 5:** Compartments of: humans (*H*) and rodents (*M*). x∈{S,D} and y∈{S,E,I,R}.

	Destination					
**Origin**	**Urban**		**R. Population**		**R. Non-Population**	
	**(u)**		**(a)**		**(f)**	
Urban (*u*)	$H_{uu}^{S}$	−	$H_{au}^{x}$	−	$H_{fu}^{x}$	−
Rural population (*a*)	$H_{ua}^{S}$	−	$H_{aa}^{x}$	$M_{aa}^{y}$	$H_{fa}^{x}$	$M_{fa}^{y}$
Rural non-population(*f*)	−	−	−	$M_{af}^{y}$	−	$M_{ff}^{y}$

Note that the reading of the subscripts is from right to left.

**Table 6:** Definition of parameters to be used in the model.

Parameter	Definition	Value *
Humans		
$\beta_{a}$	Infection rate of the mouse to the human in the populated rural sector	0.00003
$\beta_{f}$	Infection rate of mice to humans in the rural non-populated sector	0.00004
*d*	Mortality rate by disease	0.3
$\nu_{u}$	Exit rate from the urban sector	0.09
$\nu_{a}$	Departure rate from the populated rural sector	0.04
$\alpha_{au}$	Fraction of the urban population that chooses the populated rural sector as their destination	0.88
$\alpha_{fu}$	Fraction of the urban population that chooses the non-populated rural sector as their destination	0.12
$\alpha_{ua}$	Fraction of the rural population that chooses the urban sector as their destination	0.7
$\alpha_{fa}$	Fraction of the rural populated population that chooses the non-populated sector as their destination	0.04
$\tau_{u}$	Average time of an urban person employed in another sector	5
$\tau_{a}$	Average time of a person from the populated rural sector employed in another sector	5
Rodents		
β	Infection rate among rodents	0.3
γ	Recovery rate towards the disease	0.2
δ	Transition rate from State *E* to State *I*	0.02
ϕ	Rate of exit from the rural non-populated sector to the rural populated sector	0.01
$\lambda_{j}$	Percentage of the population leaving the rural non-populated sector according to their condition to the disease j∈{s,e,i,r}	0.25
σ	Average time a rodent from the rural non-populated sector remains an outsider in the rural populated sector	7
$b_{ma}$	Birth rate in the populated rural sector	0.00139
$b_{mf}$	Birth rate in the non-populated rural sector	0.00139
$d_{m}$	Mortality rate	0.00139

* Base line extracted from other investigations [21,23,26,29].

## Data Availability

Not applicable.

## Abbreviations

The following abbreviations are used in this manuscript:

HCPS	Hantavirus cardiopulmonary syndrome
SNV	Sin Nombre virus
ANDV	Andes virus
HFRS	Hemorrhagic fever with renal syndrome
HI	Hantavirus infection
PAHO	Pan American Health Organization
